# The Patterns and Drivers of Bacterial and Fungal β-Diversity in a Typical Dryland Ecosystem of Northwest China

**DOI:** 10.3389/fmicb.2017.02126

**Published:** 2017-11-10

**Authors:** Jianming Wang, Tianhan Zhang, Liping Li, Jingwen Li, Yiming Feng, Qi Lu

**Affiliations:** ^1^College of Forestry, Beijing Forestry University, Beijing, China; ^2^Institute of Remote Sensing and Digital Earth, Chinese Academy of Sciences, Beijing, China; ^3^Institute of Desertification Studies, Chinese Academy of Forestry, Beijing, China

**Keywords:** dryland, environmental selection, dispersal limitation, β-diversity, bacteria, fungi, soil microbes, community ecology

## Abstract

Dryland ecosystems cover more than 30% of the terrestrial area of China, while processes that shape the biogeographic patterns of bacterial and fungal β-diversity have rarely been evaluated synchronously. To compare the biogeographic patterns and its drivers of bacterial and fungal β-diversity, we collected 62 soil samples from a typical dryland region of northwest China. We assessed bacterial and fungal communities by sequencing bacterial 16S rRNA gene and fungal ITS data. Meanwhile, the β-diversity was decomposed into two components: species replacement (species turnover) and nestedness to further explore the bacterial and fungal β-diversity patterns and its causes. The results show that both bacterial and fungal β-diversity were derived almost entirely from species turnover rather than from species nestedness. Distance-decay relationships confirmed that the geographic patterns of bacterial and fungal β-diversity were significantly different. Environmental factors had the dominant influence on both the bacterial and fungal β-diversity and species turnover, however, the role of geographic distance varied across bacterial and fungal communities. Furthermore, both bacterial and fungal nestedness did not significantly respond to the environmental and geographic distance. Our findings suggest that the different response of bacterial and fungal species turnover to dispersal limitation and other, unknown processes may result in different biogeographic patterns of bacterial and fungal β-diversity in the drylands of northwest China. Together, we highlight that the drivers of β-diversity patterns vary between bacterial and fungal communities, and microbial β-diversity are driven by multiple factors in the drylands of northwest China.

## Introduction

The concept of β-diversity is to describe the dissimilarity in species composition among different sites, and one of the fundamental components of species diversity (Whittaker, 1960). The large-scale biogeographic pattern of β-diversity and its determinants have long been a conundrum in biogeography and ecology (Gaston et al., 2007; Lozupone and Knight, 2007; Tedersoo et al., 2014; Xia et al., 2016). Soil microbes play vital roles in maintaining the functioning of terrestrial ecosystems through regulating a series of key processes, including nutrient and material cycling (Rillig and Mummey, 2006; Bardgett et al., 2008; Handa et al., 2014). Understanding the essential processes that underlie soil microbe geographic patterns is critical in predicting ecosystem responses to global environmental change. The biogeographic patterns of microbial β-diversity and its drivers across large scales have been broadly documented (Fierer and Jackson, 2006; Fierer, 2008; Chu et al., 2010; Fierer et al., 2011; Griffiths et al., 2011; Tedersoo et al., 2014), and they have been shown to be mainly affected by environmental factors (i.e., climate, soil pH and nutrients (Prober et al., 2014 and Chen et al., 2017) and dispersal limitation (geographic distance; Wang et al., 2015; Jiang et al., 2016). However, the relative importance of these drivers to microbial β-diversity patterns might vary across geographic scale and habitat types (Bardgett and van der Putten, 2014; Wang et al., 2017); for instance, geographic distance is dominant at large scales, while environmental selection is more important at small scales (Wu et al., 2013).

The dryland region (i.e., arid, semi-arid, and dry-subhumid ecosystems) of northwest China covers a continual natural vegetation gradient from desert to meadow steppe, occupying more than 30% of the terrestrial area of China. These ecosystems are also particularly susceptible to global climate change and desertification. It has been reported that the dryland ecosystems are expanding as a result of global warming (Dai, 2013) and simultaneously extreme weather events frequently occurrence (Easterling et al., 2000). Such changes may result in the substantial changes on microbial community assembly structures (Maestre et al., 2015; Vargas-Gastelum et al., 2015). Hence, exploring the mechanism that shaped the biogeographic patterns of microbial β-diversity in dryland could provide additional understanding for maintaining biodiversity. Although many studies have explored the biogeographic patterns of microbial β-diversity in the drylands of northwest China (Wang et al., 2015; Chen et al., 2017; Wang et al., 2017), these studies mainly focused on one microbial domain and rarely concentrate on whether the biogeographic patterns are significantly different among different microbial communities.

As the two most essential taxa with interactions in the underground microorganisms, soil bacteria and fungi need to contend for similar resources (Rousk et al., 2008), but soil fungi can decompose the complex molecules in plant litter that are inaccessible for bacteria (Boer et al., 2005; Romani et al., 2006). Furthermore, the size of bacterial individuals was much smaller than fungal individuals. These differences between bacteria and fungi may results in the different response of these two microbial communities to the same ecological processes (Nielsen et al., 2010). It has been proved that bacterial and fungal communities displayed different biogeographic patterns in some regions (Zinger et al., 2011; Ma et al., 2017), and the driving factors vary between fungal and bacterial communities. For example, the environmental factors such as soil or climate plays a dominant role in the distribution of bacterial communities (Liu et al., 2014; Wang et al., 2017), while the distribution of fungal communities is better predicted by geographic distance (Jiang et al., 2016). Of course, these differences in β-diversity may be artifacts of differences in sampling scale and habitat types (Martiny et al., 2011; Wang et al., 2017). Hence, it is necessary that compare biogeographic patterns of the bacterial and fungal β-diversity based on consistently sampling and analytical methods. Despite the large number of studies, however, the processes that shaped the biogeographical patterns of bacteria and fungi have rarely been evaluated synchronously in dryland of northwest China.

Total β-diversity can be decomposed into two components: species replacement (species turnover) and species nestedness (Baselga, 2010; Legendre, 2014). Species replacement reflects the species turnover along spatial or environmental gradients, while species nestedness represents the non-random process of species gain or loss (Baselga, 2010, 2013). Many studies have shown that although both components contribute to total β-diversity, their relative roles may vary across biological groups (Baselga, 2010; Si et al., 2015). Hence, partitioning β-diversity into two components may shed new insights for exploring the bacterial and fungal β-diversity patterns and its causes. However, the relative contribution of species replacement and nestedness components to microbial β-diversity have seldom been quantified.

To compare the biogeographic patterns and the drivers of bacterial and fungal β-diversity, we collected 62 soil samples from a typical dryland region of northwest China. Soil bacterial and fungal communities were assessed base on the sequencing data of the bacterial 16S rRNA gene (V3–V4 hypervariable region) and fungal ITS regions on an Illumina MiSeq. Specifically, we addressed the following questions: (1) what is the relative contribution of species turnover and nestedness to bacterial and fungal β-diversity; (2) whether the biogeographic patterns of bacterial and fungal β-diversity are different or not? (3) if so, what causes this difference, environmental selection or dispersal limitation?

## Materials and Methods

### Study Region and Field Sampling

**FIGURE 1:**
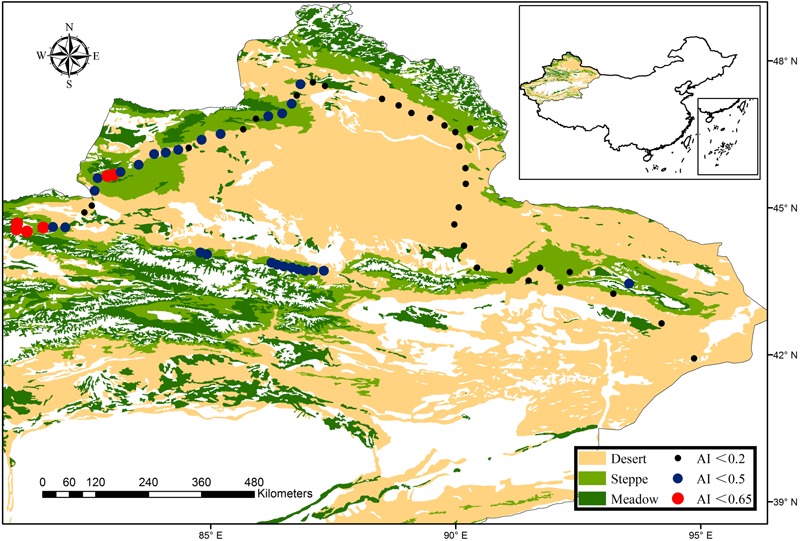
Locations of the sampling sites in a typical dryland ecosystem of northwest China. Our sampling scheme was designed to explore the patterns of bacterial and fungal β-diversity, which spans dry-subhumid, semi-arid and arid ecosystems, as demonstrated in the legend. The vegetation dataset was provided by the Data Center for Resources and Environmental Sciences, Chinese Academy of Sciences (RESDC) (http://www.resdc.cn), and the maps were created using ArcGIS 10 (http://www.esri.com/software/arcgis).

In 2016, we selected 62 sites from a typical dryland ecosystem, (the region included dry-subhumid, semi-arid and arid ecosystems; United Nations Environment Programme, 1992; **Figure 1**) in the northern Xinjiang Uygur Autonomous Region (41° 56′ N to 47° 34′ N and 81° 2′ E to 94° 52′ E) during the peak growing season (July–August). The sampling sites have high precipitation and temperature variability, the mean annual precipitation ranged from 43 to 458 mm, mean annual temperature was between -0.6 and 9.0°C, and aridity index (AI) ranged from 0.04 to 0.64. The sampling region mainly comprised three vegetation types that changed from desert, desert steppe to typical steppe with increasing altitude.

At each site, a 100 m^2^ plot was established in an area with typical dryland vegetation, and geographical coordinates (latitude and longitude) and altitude of each plot were recorded using a GPS. At each plot, soil samples were collected from 15 randomly selected points (0–10 cm depth) in vegetated areas and then mixed together into a single sample. The well-mixed soil sample was sieved through 2 mm mesh and then subdivided into two portions: the first portion was stored in thermal insulated boxes (at 4°C) for determining the soil physicochemical properties, and the other portion was stored at -20°C before DNA extraction.

### Soil Physicochemical Properties

Soil total nitrogen (TSN) and total organic carbon (TOC) were determined using the Kjeldahl procedure (Michałowski et al., 2013) and K2Cr2O7 oxidation method (Walkley, 1947), respectively. Soil total phosphorus (TSP) was measured using the molybdenum blue method (Kuo, 1996) and soil available nitrogen (AN) was determined by the Alkali diffusion method. The ratios of N: P and C: N were calculated. Soil water saturation of total water holding capacity (TW) was measured gravimetrically after drying soil in an oven at 105°C for 48 h. Finally, soil pH was determined in a 1: 2.5 ratio of fresh soil to water slurry.

### Climate Data

Climatic variables, including mean annual temperature (MAT) and mean annual precipitation (MAP), were extracted from WorldClim global climate database using the geographic coordinates for each site^1^. We then obtained annual potential evapotranspiration (PET) data from CGIAR-CSI^2^. AI was estimated as the ratio of MAP to PET (after United Nations Environment Programme, 1992).

### DNA Extraction, PCR Amplification, and Illumina-Based Sequencing

Genomic DNA was extracted from the 0.5 g fresh soil samples using E.Z.N.A. soil DNA kits (OMEGA, United States) following the manufacturer’s instructions. All extracted DNA samples were stored at -20°C for subsequent analysis.

To assess the bacterial and fungal community composition, we amplified the V3-V4 hypervariable region of the bacterial 16S rRNA gene, using the forward primer 338F (5′-ACTCCTACGGGAGGCAGCAG-3′) and the reverse primer 806R (5′-GGACTACHVGGGTWTCTAAT-3′), and the fungal ITS regions, using the forward primer ITS1-F (5′-CTTGGTCATTTAGAGGAAGTAA-3′) and the reverse primer ITS2 (5′-TGCGTTCTTCATCGATGC-3′). These primers contained a set of 8-nucleotide barcode sequences unique to each sample. PCR amplifications were performed following the procedure described by Miao et al. (2016).

PCR products were pooled and purified using the Agarose Gel DNA purification kit (Axygen Biosciences, Union City, CA, United States). The purified PCR products were pooled in equimolar concentrations and paired-end sequenced (2 × 300) on an Illumina MiSeq platform according to the standard protocols.

### Bioinformatics Analysis

We processed the high-quality sequence data in the QIIME package (Quantitative insights into microbial ecology; v1.2.1), according to the procedure described by Fierer et al. (2008), He et al. (2010), and Miao et al. (2016). The unique sequence set was classified into operational taxonomic units (OTUs) based on the threshold of 97% identity using UCLUST. Chimeric sequences were identified and removed using Usearch (version 8.0.1623). The taxonomy of each 16S rRNA gene sequence was analysed against the Silva119 16S rRNA database using UCLUST with a confidence threshold of 90%, while the taxonomy of each ITS gene sequence was analyzed by comparison against sequences within the Unite 7.0 database using UCLUST.

The bacterial and fungal DNA sequences in our study have been submitted in SRA of NCBI database under accession number SRP119963 and SRP119964, respectively.

### Statistical Analyses

First, the determinant phyla of bacterial and fungal communities with a relative abundance greater than 0.5% were compiled, and the association between these determinant phyla and environmental variables were calculated using Spearman’s rank correlation analyses. The overall variations in fungal and bacterial communities were characterized by non-metric multidimensional scaling (Kruskal, 1964).

Second, we estimated the pairwise geographic distance using the fossil package according to the GPS coordinates. For the bacterial and fungal abundance data with less extreme distributions, square root transformation was used prior to calculation (Legendre, 2014). Pairwise environmental distances (Euclidean distance) and pairwise community Bray-Curtis distance (community dissimilarity, that is β-diversity) between sites were calculated within the “vegan package” (Oksanen et al., 2016) and β-diversity (d_BC_) was separated into species turnover (d_BC-bal_) and nestedness (d_DC-gra_) within the “betapart” package (Baselga and Orme, 2012).

Mantel tests (10,000 permutations) were used to explore the significance of the influence of geographic and environmental distances on total β-diversity and its components. We also used partial Mantel tests (10,000 permutations) to separate the influences of geographic distance and environmental distances on total β-diversity and its components (Martiny et al., 2006).

Distance-decay curves not only provide a quantitative analysis for the variability in community composition among different sites (that is, β-diversity; Anderson et al., 2011), but also the distance-decay slope can directly reflect the species turnover rate over geographic zones (Wang et al., 2017). Therefore, the slope of least squares regression of the relationship between ln-transformed community similarity and ln-transformed geographic distance (Martiny et al., 2011) was used to calculate the distance–decay rate of the microbial communities. We then examined whether the species turnover rate of the distance-decay curve (least squares) was significantly different between bacterial and fungal communities or different from zero by using the matrix permutations, based on 10,000 permutations (Nekola and White, 1999).

Finally, we conducted a multiple regression on matrices (MRM) approach to examine the relative impacts of geographical distance versus environmental variables on β-diversity and its two components (Legendre et al., 1994; Lichstein, 2007). To avoid strong collinearity between the variables, we used the varclus procedure to evaluate the redundancy of the variables in the Hmisc R package before running MRM (after Wang et al., 2017); we then removed TSN and NP because of Spearman’s ρ^2^ > 0.7 (Supplementary Figure S2), and all the other variables were entered into the final model. We conducted the standardized predictor variables and matrix randomization procedure in the ecodist R package (Goslee and Urban, 2007). To prevent the influence of data overfitting, we ran the first MRM test to remove the non-significant variables (Wang et al., 2017), and then re-ran the MRM test. We report the model results of this second run.

## Results

### Bacterial and Fungal Community Composition

Our studies identified a total of 1,136,292 high-quality bacterial sequences and 23,50,669 high-quality fungal sequences from 62 soil samples that were grouped into 5,532 bacterial OTUs and 5,788 fungal OTUs, respectively (Supplementary Figure S1 and Supplementary Table S1). The range of bacterial sequences per sample was from 10,476 to 28,292 (with an average of 18,327 ± 3,788 sequences, Supplementary Table S1) that were classified as 1,002 to 1,922 OTUs (with an average of 1,482 ± 210 OTUs). Further, the fungal sequences per sample changed from 19,581 to 45,083 (with an average of 37,914 ± 6,556) that were classified as 342 to 1,081 OTUs (with an average of 765 ± 170 OTUs, Supplementary Table S1).

**FIGURE 2:**
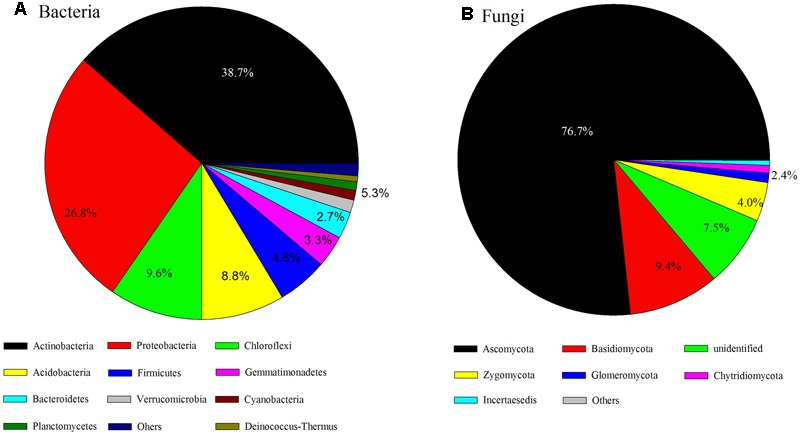
Bacterial **(A)** and fungal **(B)** taxonomic composition and their variations over sampling sites.

The dominant phyla of bacterial communities across all soil samples were Actinobacteria, Proteobacteria, Chloroflexi, Acidobacteria, Firmicutes, Gemmatimonadetes, Bacteroidetes, Verrucomicrobia, Cyanobacteria, Planctomycetes, and Deinococcus-Thermus (the relative abundance > 0.5%, **Figure 2A**) and these dominant phyla occupied more than 90% of the bacterial sequences. The fungal communities were dominated by Ascomycota, Basidiomycota, unidentified fungi, Zygomycota, Glomeromycota, Chytridiomycota (**Figure 2B**); more than 90% of the fungal sequences belonged to these dominant phyla.

The relative abundance of some bacterial and fungal phyla was remarkably correlated with aridity and soil attributes (**Tables 1, 2**). The relative abundance of Acidobacteria (*R* = 0.57, *P* < 0.01), Verrucomicrobia (*R* = 0.66, *P* < 0.01), Zygomycota (*R* = 0.53, *P* < 0.01) and Glomeromycota (*R* = 0.56, *P* < 0.01) were positively correlated with AI, whereas the abundance of Deinococcus-Thermus (*R* = -0.58, *P* < 0.01) and Incertae sedis (*R* = -0.59, *P* < 0.01) were negatively correlated with AI. Furthermore, soil pH, water saturation of total water holding capacity (TW), total organic carbon (TOC), total nitrogen (TSN), carbon/nitrogen ratio (CN) and Altitude also were significantly correlated with many dominant bacterial and fungal taxa (see **Tables 1, 2**).

**Table 1:** Spearman’s rank correlation coefficients between dominant bacterial taxa and environmental variables.

	TW	TSN	TSP	TOC	AN	pH	CN	NP	AI	Altitude
Actinobacteria	-0.34**	-0.11	0.14	-0.13	-0.29*	0.31*	-0.22	-0.14	-0.03	-0.25*
Proteobacteria	-0.08	-0.01	0.02	0.04	0.15	-0.05	0.23	-0.02	-0.17	-0.07
Chloroflexi	-0.28*	-0.28*	0.14	-0.25	-0.34**	0.302*	-0.09	-0.32*	-0.21	-0.11
Acidobacteria	0.50**	0.61**	0.05	0.66**	0.36**	-0.55**	0.19	0.64**	0.57**	0.57**
Firmicutes	-0.03	-0.16	-0.29*	-0.21	0.11	-0.01	0.03	-0.13	-0.22	-0.09
Gemmatimonadetes	0.18	0.18	0.06	0.18	0.2	-0.11	0.08	0.13	0.21	0.23
Bacteroidetes	0.18	-0.06	-0.08	0.03	0.07	0.04	0.28*	-0.02	-0.11	0.03
Verrucomicrobia	0.45**	0.54**	0.03	0.60**	0.26*	-0.38**	0.09	0.58**	0.66**	0.38**
Cyanobacteria	0.16	0.26*	-0.12	0.31*	0.22	-0.19	0.07	0.30*	0.17	0.28*
Planctomycetes	0.10	0.11	0.01	0.17	-0.01	-0.05	-0.01	0.15	0.21	-0.02
Deinococcus-Thermus	-0.55**	-0.67**	-0.11	-0.68**	-0.36**	0.64**	-0.14	-0.64**	-0.57**	-0.59**

*TW, water saturation of total water holding capacity; AN, soil available nitrogen; TOC, soil total organic carbon; TSN, soil total nitrogen; NP, nitrogen/phosphorus; TSP, soil total phosphorus; CN, carbon/nitrogen; AI, aridity index; and, ^∗∗^*P* < 0.01, ^∗^*P* < 0.05.*

**Table 2:** Spearman’s rank correlation between dominant fungal taxa and environmental variables.

	TW	TSN	TSP	TOC	AN	pH	CN	NP	AI	Altitude
Ascomycota	-0.38**	-0.22	-0.09	-0.23	0.03	0.2	-0.13	-0.18	-0.16	-0.47**
Basidiomycota	0.09	0.05	0.22	0.07	-0.15	0.03	0.11	-0.01	0.08	0.28*
Unidentified	0.13	-0.04	-0.12	-0.09	-0.31*	0.19	-0.14	-0.02	-0.07	0.05
Zygomycota	0.68**	0.72**	0.04	0.77**	0.45**	-0.49**	0.15	0.73**	0.53**	0.61**
Glomeromycota	0.41**	0.61**	0.09	0.56**	0.22	-0.27*	-0.19	0.60**	0.56**	0.36**
Chytridiomycota	-0.01	-0.04	0.09	-0.01	-0.13	-0.06	-0.07	-0.03	-0.06	-0.21
Incertae sedis	-0.30*	-0.53**	-0.26*	-0.54**	-0.44**	0.47**	0.03	-0.50**	-0.59**	-0.34**

*TW, soil water saturation of total water holding capacity; AN, soil available nitrogen; TOC, soil total organic carbon; TSN, soil total nitrogen; NP, nitrogen/phosphorus; TSP, soil total phosphorus; CN, carbon/nitrogen; AI, aridity index; and, ^∗∗^*P* < 0.01, ^∗^*P* < 0.05.*

### Bacterial and Fungal Community Structure and β-Diversity

**FIGURE 3:**
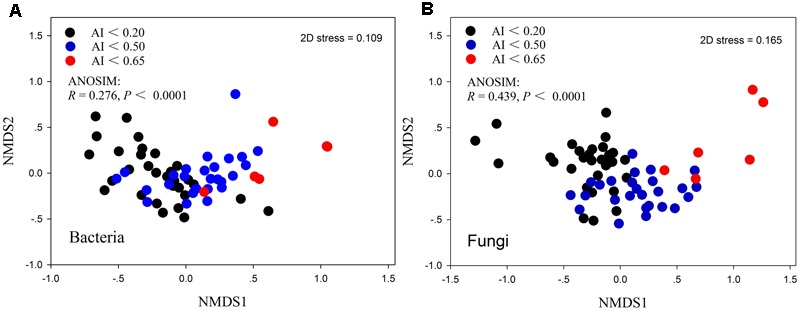
Non-metric multidimensional scaling (NMDS) ordination of the bacterial community **(A)** and fungal community **(B)** compositions from 62 samples.

The patterns of bacterial and fungal community composition across all samples were visualized using non-metric multidimensional scaling ordination based on the Bray-Curtis distances (**Figure 3**). The dissimilarity analysis of community composition revealed that both fungal and bacterial community structures were markedly varied across the AI gradients (*R* = 0.276 and 0.439, *P* < 0.0001, respectively; **Figures 3A,B**). Mantel correlation analyses showed that the bacterial and fungal community structures were significantly correlated with selected environmental factors, whereas were stronger influenced by AI, TW, TOC, TAN, pH (**Table 3**).

**Table 3:** The environmental variables that were significantly correlated with bacterial and fungal community structure.

	Bacteria		Fungi	
	*r*	*P*	*r*	*P*
TW	**0.343**	***P* < 0.0001**	**0.305**	***P* < 0.001**
TSP	0.253	*P* < 0.001	0.164	*P* < 0.01
TSN	**0.356**	***P* < 0.0001**	**0.403**	***P* < 0.0001**
NP	0.292	*P* < 0.0001	0.383	*P* < 0.0001
CN	0.251	*P* < 0.001	0.281	*P* < 0.001
TOC	**0.354**	***P* < 0.0001**	**0.384**	***P* < 0.0001**
AN	0.216	*P* < 0.01	0.257	*P* < 0.001
pH	**0.402**	***P* < 0.0001**	**0.343**	***P* < 0.0001**
AI	**0.382**	***P* < 0.0001**	**0.581**	***P* < 0.0001**
Altitude	0.241	*P* < 0.0001	0.291	*P* < 0.0001

*The correlation (*r*) and significance (*P*) were determined by Mantel tests based on 10,000 permutations between community structure (Bray-Curtis dissimilarity) and environmental variables (Standardized Euclidean distance). Values in bold indicate relative stronger correlation (*r*) in both bacterial and fungal communities.*

**FIGURE 4:**
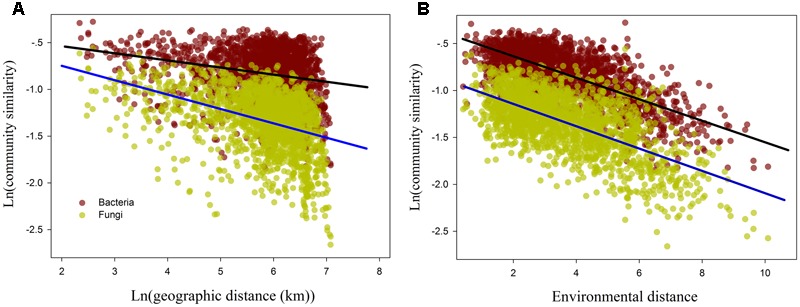
The relationship between the bacterial and fungal β-diversity and ln geographic distance **(A)** and environmental distance **(B)**. The regression line of **(A)** denotes distance-decay curves for the bacterial (black line) and fungal (blue line) communities.

The relationship between ln-transformed geographic distance versus ln-transformed community similarity (1-Bray-Curtis index) revealed a significant distance–decay relationship for fungal and bacterial samples (*P <* 0.001; **Figure 4A** and Supplementary Table S2). Furthermore, the slope of the distance–decay relationship between fungal and bacterial communities estimated by least squares regression models were significantly less than zero (*P <* 0.0001). However, the distance-decay slopes of fungal communities (slope = -0.152, *P <* 0.0001; **Figure 4A** and Supplementary Table S2) were significantly steeper than that of bacterial communities (slope = -0.074, *P <* 0.001; **Figure 4A** and Supplementary Table S2). Moreover, the overall community similarity (average value = 0.242, Supplementary Table S2) in fungal community composition was markedly lower than bacterial community similarity (average similarity = 0.454, Supplementary Table S2).

When both components of β-diversity were decomposed, the species turnover almost entirely explained the bacterial and fungal (94.86 and 95.77%, respectively; **Table 4**) β-diversity rather than nestedness (5.41 and 4.41%, respectively). We also found that β-diversity and species turnover of bacteria and fungi significantly increased with increasing geographic distance (*P <* 0.0001; Supplementary Figure S3), where the slope for fungal β-diversity and species turnover was markedly steeper than that for bacterial β-diversity and species turnover. Furthermore, both bacterial and fungal nestedness showed no significant response to geographic distance (*P >* 0.05; Supplementary Figure S3).

**Table 4:** Summary statistics of the bacterial and fungal β-diversity, as measured with the Bray-Curtis dissimilarity (d_BC_) and its replacement (d_BC-bal_) and nestedness (d_BC-gra_) component.

	Bacteria			fungi		
	d_BC_	d_BC-bal_	d_BC-gra_	d_BC_	d_BC-bal_	d_BC-gra_
Mean value	0.545	0.517	0.028	0.757	0.725	0.032
Relative contribution (%)		94.862	5.416		95.773	4.414

*d_*B*C_, β-diversity; d_*BC*-*bal*_, replacement component; d_*BC*-*gra*_, nestedness component.*

The results of the Mantel test showed that environmental distance was strongly correlated with β-diversity and species turnover of bacterial and fungal communities (*P <* 0.0001; **Figure 4** and **Table 5**). The partial-Mantel test revealed that when geographic distance was controlled, both β-diversity and species turnover of bacterial and fungal communities were still significantly correlated with environmental distance (*P <* 0.001, respectively; **Table 5**). Geographical distance was also significantly related to β-diversity and species turnover in bacterial and fungal communities (*P <* 0.0001; **Figure 4** and **Table 5**). When controlling for environmental distance, however, geographic distance was not significantly related to β-diversity and species turnover for bacterial communities (*P >* 0.05; **Table 5**). In contrast, geographic distance had a significantly independent influence on fungal β-diversity and species turnover (*P* < 0.0001; **Table 5**). Moreover, both bacterial and fungal nestedness showed no significant response to geographic and environmental distance.

**Table 5:** The relationship between community dissimilarity and the environmental distance or geographic distance using Mantel and partial Mantel tests.

Effects of	Controlling for	Bacteria			Fungi		
		d_BC_	d_BC-bal_	d_BC-gra_	d_BC_	d_BC-bal_	d_BC-gra_
Environmental distance		0.636***	0.628***	-0.091^NS^	0.621***	0.552***	0.032^NS^
Geographic distance		0.221***	0.209***	0.027^NS^	0.428***	0.415***	-0.032^NS^
Environmental distance	Geographic distance	0.612***	0.606***	-0.104^NS^	0.568***	0.489***	0.045^NS^
Geographic distance	Environmental distance	**0.032**	**0.019**	0.058^NS^	**0.316^∗∗∗^**	**0.308^∗∗∗^**	-0.041^NS^

*d_*BC*_, β-diversity; d_*BC*-*bal*_, replacement component; d_*BC*-*gra*_, nestedness component. ^∗^*P* < 0.01, ^∗∗^*P* < 0.001, and ^∗∗∗^*P* < 0.0001; NS *P* > 0.05. Values in bold highlight the different response of bacterial and fungal communities to geographic distance.*

### The Relative Roles of Geographic Distance and Environmental Factors in Determining the Fungal and Bacterial β-Diversity

**FIGURE 5:**
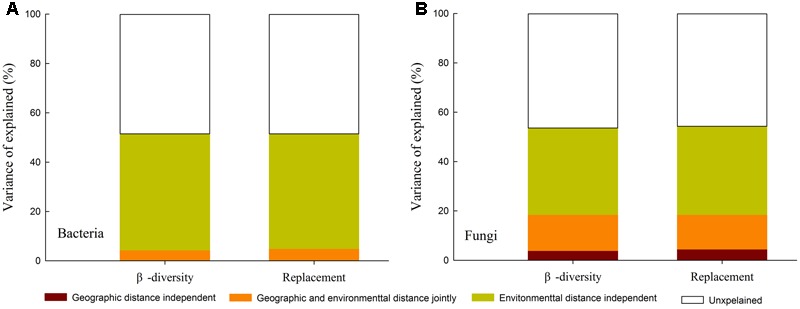
Variation partitioning for the effects of geographical distance versus environmental factors on β-diversity and replacement (turnover) component of bacterial **(A)** and fungal communities **(B)**.

A MRM was conducted to further demonstrated the relative influence of geographic distance and environmental factors on β-diversity and its components. The results showed that environmental variables could independently explain a significant and large proportion of the variance in the β-diversity and species turnover for bacterial (*R*^2^ = 0.471 and 0.465, respectively; **Figure 5A**) and fungal communities (R^2^ = 0.351 and 0.358, respectively; **Figure 5B**). Geographic distance was retained into the final model of fungal β-diversity and species turnover, and could individually explain 3.9 and 4.5% variance of fungal β-diversity and species turnover, respectively. However, geographic distance was excluded from the final model for bacterial β-diversity and species turnover (*P* > 0.05). Among the environmental factors, TW, TSP, pH, CN and AI were retained in the models of second runs, and TW and pH were relatively more important in explaining bacterial β-diversity and species turnover (partial regression coefficient *b* = 0.035 (0.036) and 0.036 (0.037); *P* < 0.0001, respectively). Moreover, AI and ln [Geographic distance (km)] contributed the relatively larger partial regression coefficient in explaining fungal β-diversity and species turnover [*b* = 0.032 (0.027) and 0.019 (0.022), *P* < 0.0001 and 0.01, respectively; **Table 6**].

**Table 6:** Results of the multiple regression analysis on matrices analysis (MRM) for bacterial and fungal communities.

	Bacteria		Fungi	
	*R*^2^= 0.515	*R*^2^= 0.515	*R*^2^= 0.536	*R*^2^= 0.543
	β-diversity	Replacement	β-diversity	Replacement
Ln (Geographic distance (km))			**0.019^∗∗∗^**	**0.022^∗∗∗^**
SM	**0.035^∗∗∗^**	**0.036^∗∗∗^**		
TSP	0.015*	0.015*		
TOC				0.018**
AN				
pH	**0.036^∗∗∗^**	**0.037^∗∗∗^**	0.016**	0.009*
CN	0.029***	0.029**	0.017**	0.018**
AI	0.028**	0.029***	**0.032^∗∗∗^**	**0.027^∗∗∗^**
Altitude				

*d_*BC*_, β-diversity; d_*BC*-*bal*_, replacement component; d_*BC*-*gra*_, nestedness component; TW, water saturation of total water holding capacity; TOC, soil total organic carbon; TSP, soil total phosphorus; CN, carbon/nitrogen; AI, aridity index. *R*^2^ represents the variation of β-diversity or replacement component. The partial regression coefficients and associated *P*-values of the final model are reported from permutation test (nperm = 10,000) if its significance level is 0.05. ^∗^*P* < 0.01, ^∗∗^*P* < 0.001, and ^∗∗∗^*P* < 0.0001. Values in bold indicate the relatively more important factors for bacterial and fungal communities, respectively.*

## Discussion

Exploring the biogeographic pattern of different microbial communities is crucial to understand the underlying mechanisms that shape microbial diversity (Martiny et al., 2006; Chen et al., 2016). It is widely reported that microbial community similarity is negatively related to geographic distance (Morlon et al., 2008; Jiang et al., 2016; Wang et al., 2017). The distance-decay curve can directly reflect the variation in the community species similarity with increasing geographic distance (Green and Bohannan, 2006). Moreover, when geographic distance was used as independent variable, the rate of decreasing community similarity was equivalent to the rate of species turnover between communities (Nekola and White, 1999). From our results, both bacterial and fungal community similarity significantly decreased with increasing geographic distance (*P* < 0.001; **Figure 5A** and Supplementary Table S2), indicating that the obvious biogeographic patterns of the fungal and bacterial β-diversity across the drylands region of northwest China. Both the distance decay patterns of fungal and bacterial communities are consistent with previous observations in the dryland regions (Maestre et al., 2015; Wang et al., 2015 Cao et al., 2016; Chen et al., 2017). Some studies have showed that bacterial and fungal communities display different biogeographic patterns in other regions (Zinger et al., 2011; Jiang et al., 2016; Ma et al., 2017). In our study, we found that the slope of the distance-decay curve of the fungal communities was significantly steeper than that of the bacterial communities, which is consistent with the findings of Jiang et al. (2016) and indicate that the species turnover rate of the fungal communities is distinctly faster than that of the bacterial communities. Furthermore, geographic distance explained 18.32% of the variation in the soil fungal community, but only 4.84% of the variation in the bacterial community. Together, these results confirm that the biogeographic patterns of β-diversity also differ significantly between bacterial and fungal communities in the dryland of northwest China.

Environmental selection and dispersal limitation are considered to be two dominating ecological processes in controlling the biogeographic patterns of β-diversity (Ganderton and Coker, 2005; Tang et al., 2012; Wang et al., 2015; Jiang et al., 2016). However, the relative effects of these two processes on the patterns of microbial β-diversity might vary across geographic scale, habitat and taxa types (Griffiths et al., 2011; Xiong et al., 2012; Bardgett and van der Putten, 2014; Wang et al., 2015; Jiang et al., 2016). Previous studies claimed that environmental selection processes have greater influence than geographic distance in driving microbial β-diversity (Fenchel et al., 1997; Finlay, 2002). Here, we found that environmental distance can individually influence both the bacterial and fungal β-diversity (ρ = 0.612 and 0.568, *P* < 0.0001, respectively), demonstrating the dominant influence of environmental selection on the microbial β-diversity. Geographical distance can individually affect the fungal β-diversity(ρ = 0.316, *P* < 0.0001, respectively), while had no independent influence on bacterial β-diversity (*P* > 0.05). The result of the MRM model analysis further confirmed these findings, since geographic distance was only retained into the final model for the fungal communities, whereas some environmental variables were retained into the final models for bacterial and fungal communities. Therefore, environmental selection has a sole influence on the bacterial β-diversity, while the fungal β-diversity was determined by environmental selection and dispersal limitation together. These findings may suggest that environmental selection has a dominant influence on microbial β-diversity, while the role of dispersal limitation is taxa-dependent, in the drylands of northwest China. Indeed, the environmental selection process represents the difference of the relative fitness among taxonomic groups under environmental stress (Chesson, 2000), whereby such differences result in the variation of the microbial community composition across space which will strengthen with increasing environmental differentiation, and thus also can produce a significant distance–decay relationship (Wang et al., 2017). In consequence, the distance-decay patterns of bacteria may be primarily attributed to the environmental selection process, whereas those of fungi may mainly arise from the combined effects of environmental selection and dispersal limitation.

Species turnover almost entirely explained the bacterial and fungal β-diversity (94.86 and 95.77%, respectively) rather than nestedness, implying that both the bacterial and fungal β-diversity may mainly arise from species turnover component. Furthermore, the responses of β-diversity to environmental variables or geographic distance is highly consistent with that of species turnover. Species nestedness only contributed 5.41 and 4.41% of the bacterial and fungal β-diversity, respectively and did not significantly respond to geographic and environmental distance. We suggest that the influence of environmental selection and dispersal limitation on species turnover may represent the influence of these ecological processes on bacterial and fungal β-diversity in our study. Due to the influence of “body size” of bacteria and fungi (Martiny et al., 2006; Fierer, 2008), and the relatively smaller-sized bacteria may be able to protect themselves from being affected by long-term dispersal limitations (Fenchel et al., 1997; Finlay, 2002), whereas fungi have limited capacity for long distance dispersal at regional scales (Chen et al., 2017). Hence, the species turnover of fungal communities is more easily influenced by geographic distance. In our study, environmental distance can individually and strong affect the β-diversity and species turnover for both bacterial and fungal communities, indicating the dominant role of environmental selection. However, only fungal β-diversity and species turnover were independently affected by geographic distance, implying that the roles of environmental selection and dispersal limitation to species turnover vary across bacterial and bacterial communities. Such a difference in the responses of bacteria and fungi to geographic distance resulted in a faster rate of species turnover in the fungal communities of our study (Supplementary Figure S3), as illustrated by the steeper distance-decay slope of fungal communities than that of the bacterial communities.

In our study, although environmental factors such as AI, CN, pH significantly affected both bacterial and fungal β-diversity and species turnover, there was a fraction that could not be explained by the selected environmental factors. Further, the pure fraction of geographic distance also potentially reflects the influence of biotic and other unidentified factors (Borcard et al., 1992; Smith and Lundholm, 2010), in addition to dispersal limitation (Gilbert and Lechowicz, 2004). Therefore, biotic and other factors may also have an important impact on bacterial and fungal β-diversity. In consequence, we suggest that differences in the role of dispersal limitations and other, unknown, processes in bacterial and fungal species turnover may lead to the biogeographic patterns for bacterial and fungal β-diversity differ remarkably in the dryland of northwest China. Our findings highlight that the mechanisms that control the β-diversity patterns vary across taxonomic groups. Furthermore, we demonstrated that species turnover almost entirely explained the bacterial and fungal β-diversity rather than species nestedness, and so the two components of β-diversity respond differently to ecological processes may shed new insights for protecting the microbial functional diversity in dryland ecosystems.

## Conclusion

We synchronously compared the biogeographic patterns of bacterial and fungal β-diversity in a typical dryland region of northwest China base on consistently sampling and analytical methods. We quantified the relative contribution of species replacement (turnover) and nestedness components of β-diversity to microbial communities. We found that distance-decay relationships showed that β-diversity patterns of bacterial and fungal are different, and that the β-diversity derives almost entirely from species turnover rather than from species nestedness. We also found that environmental selection had the dominant influence on bacterial and fungal β-diversity and species turnover, while the role of dispersal limitation on β-diversity and species turnover varies across bacterial and bacterial communities. The different response of bacterial and fungal species turnover to dispersal limitation or other unknown processes may result in different biogeographic patterns of bacterial and fungal β-diversity in the drylands of northwest China.

## Author Contributions

JW and JL designed the study; JW, JL, and QL developed the methods; JW and TZ performed the field investigation and collected the data; JW, LL and YF conducted the analyses; JW and JL wrote the paper.

## Conflict of Interest Statement

The authors declare that the research was conducted in the absence of any commercial or financial relationships that could be construed as a potential conflict of interest.

## Footnotes

1 http://www.worldclim.org

2 http://www.cgiar-csi.org
