# Supplementary material for: The Patterns and Drivers of Bacterial and Fungal β-Diversity in a Typical Dryland Ecosystem of Northwest China
Source: Front Microbiol. 2017 Nov 10;8:2126. doi: 10.3389/fmicb.2017.02126 (PMC5686094; doi:10.3389/fmicb.2017.02126)
Supplement: Supplementary file 1 [file Data_Sheet_1.doc]

**The patterns and drivers of** **bacterial and fungal β-diversity in a typical dryland ecosystem of northwest China**

Jianming Wang^1^, Tianhan Zhang^1^, Liping Li^2^, Jingwen Li**^1*^**, Yiming Feng^3^ and Qi Lu^3^

1 The College of Forestry of Beijing Forestry University, Beijing Forestry University, No. 35 Qinghua East Road, Haidian District, Beijing 100083, China.

2 Institute of Remote Sensing and Digital Earth, Chinese Academy of Sciences, Beijing 100101, China.

3 Institute of Desertification Studies, CAF, Beijing, No.10 Huaishuju Road, Haidian District, Beijing, 100091.

E-mail: lijingwenhy@bjfu.edu.cn

* Corresponding author

Telephone number: +8601062338100. E-mail address: lijingwenhy@bjfu.edu.cn (J. Li)

Table S1 Summary statistics of the illumina sequencing results for the bacterial and fungal communities

|  | bacteria |  | Fungi |  |
| --- | --- | --- | --- | --- |
|  | Reads | OTUs | Reads | OTUs |
| Total | 1136292 | 5532 | 2350669 | 5788 |
| Mean | 18327 ± 3788 | 1482 ± 210 | 37914 ± 6556 | 765 ± 170 |

Table S2 Summary statistics of the distance-decay relationship for the bacterial and fungal communities

|  | Samples | Intercept | Slope (*z*) | *Avg* | Mantel *r* | *P* |
| --- | --- | --- | --- | --- | --- | --- |
|  |  |  |  | *similarity* |  |  |
| Bacteria | 62 | -0.396 | -0.074 | 0.454 | -0.221 | <0.0001 |
| Fungi | 62 | -0.452 | -0.152 | 0.242 | 0.428 | <0.0001 |

Statistic result of ordinary least squares regressions of ln-transformed community similarity against ln-transformed geographical distance. The Mantel statistic *r* estimates the correlation between OTU similarity matrices and geographical distance matrices. *P*-values are based on 10,000 randomized pairings of OTU similarity and geographical distance.

**Figure legends:**

**Figure S1** Rarefaction curves of the samples from bacteria (a) and fungi (b) at 97% similarity grouping of the 16S rDNA and ITS sequences, respectively. The vertical axis shows the number of OTUs that would be expected to be found after sampling the numbers of tags shown on the horizontal axis. Lines of different colors represent different sample

**Figure S2**. Cluster analysis of the measured environmental variables. The analysis was performed and plotted using varclus in Hmisc R package. Sampling points with all measurements were included (*n* = 62). AN, Soil available nitrogen; TW, Soil water saturation of total water holding capacity; TOC, Soil total organic carbon; TSN, Soil total nitrogen; NP, nitrogen/phosphorus; TSP, Soil total phosphorus; CN, carbon/nitrogen; AI, aridity index

**Figure S3**. The relationship between ln geographic distance and the bacterial (A, C, E) and fungal (B, D, F) β-diversity, as measured with the Bray-Curtis disimilarity (d_BC_) and its replacement (d_BC-bal_) and nestedness (d_BC-gra_) component. Black curves are the ﬁtted functions.
