# Supplementary figures and images for: The Patterns and Drivers of Bacterial and Fungal β-Diversity in a Typical Dryland Ecosystem of Northwest China
Source: Front Microbiol. 2017 Nov 10;8:2126. doi: 10.3389/fmicb.2017.02126 (PMC5686094; doi:10.3389/fmicb.2017.02126)

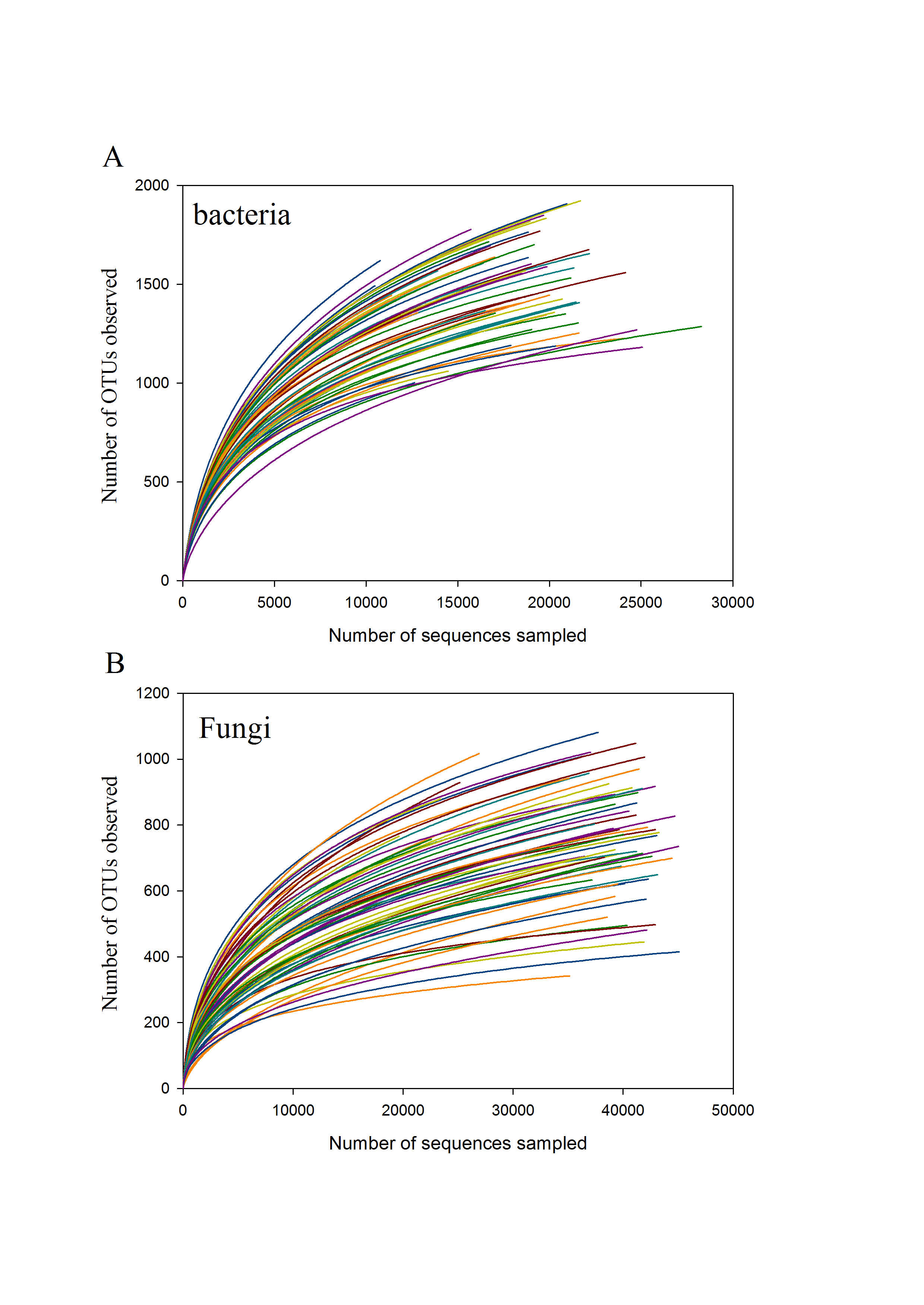

Supplement: Supplementary file 2 [file Image_1.JPEG]

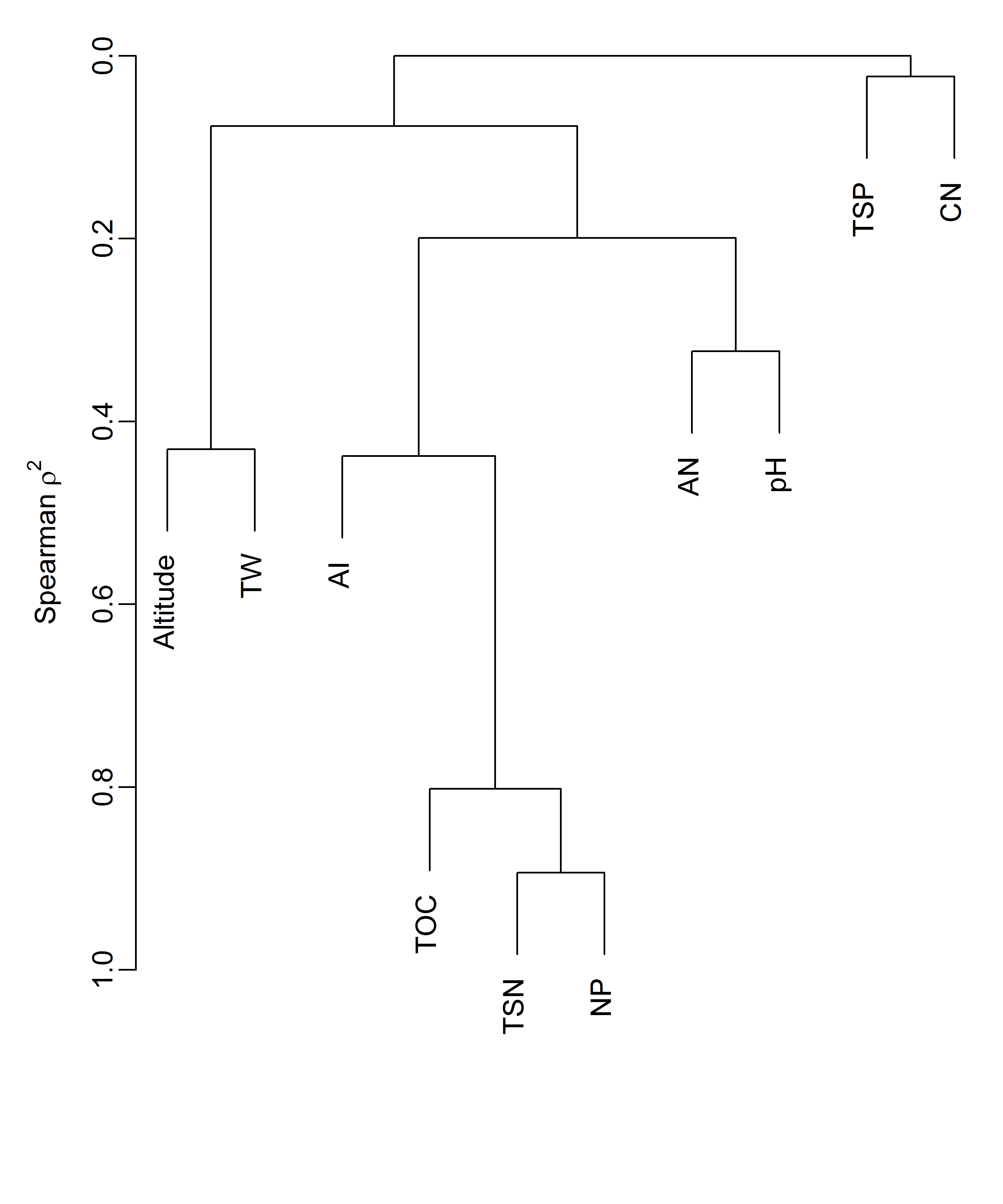

Supplement: Supplementary file 3 [file Image_2.TIF]

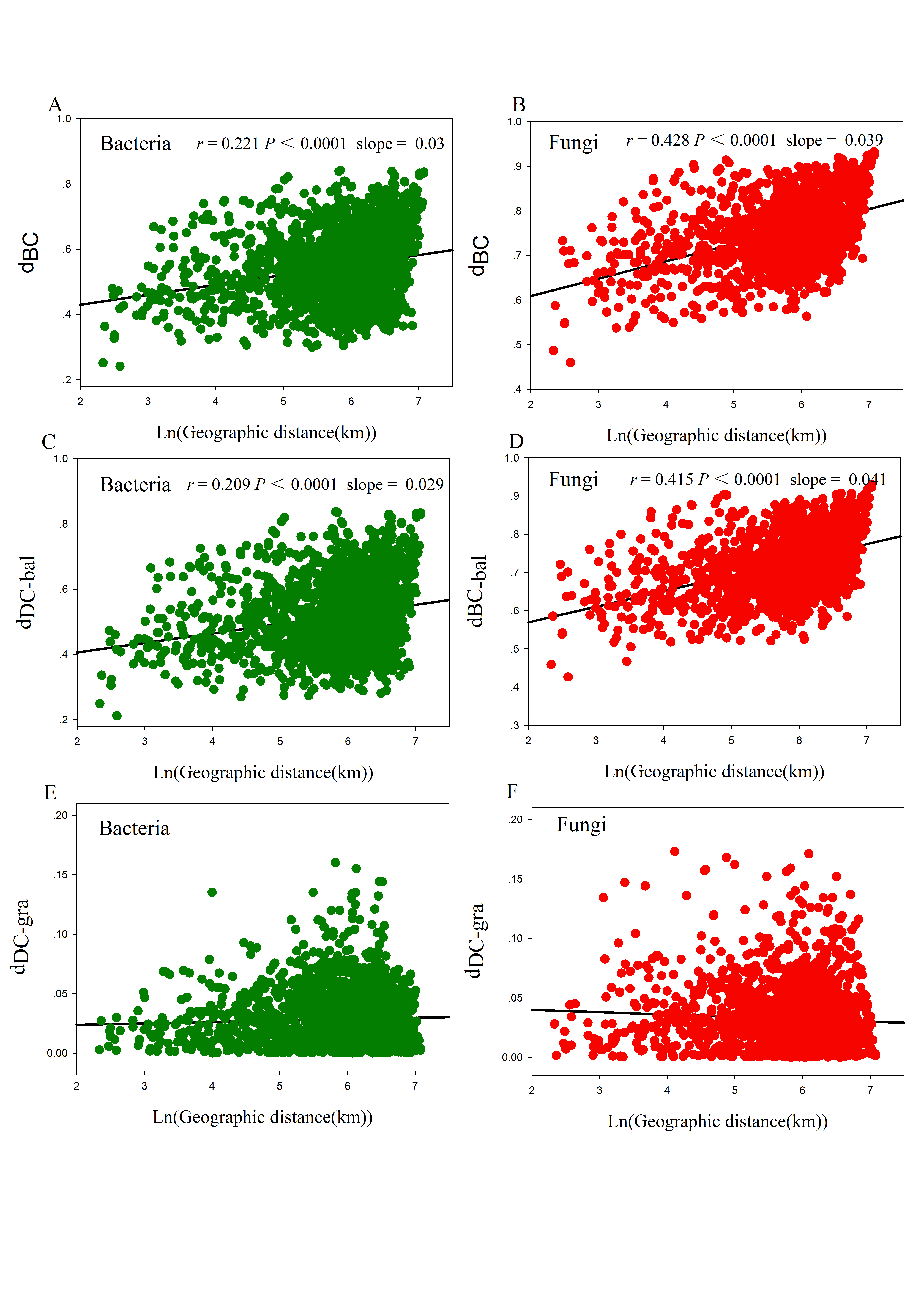

Supplement: Supplementary file 4 [file Image_3.JPEG]
